# Paired Associative Stimulation with High-Frequency Peripheral Component Leads to Enhancement of Corticospinal Transmission at Wide Range of Interstimulus Intervals

**DOI:** 10.3389/fnhum.2016.00470

**Published:** 2016-09-23

**Authors:** Anastasia Shulga, Aleksandra Zubareva, Pantelis Lioumis, Jyrki P. Mäkelä

**Affiliations:** ^1^BioMag Laboratory, HUS Medical Imaging Center, University of Helsinki and Helsinki University HospitalHelsinki, Finland; ^2^Clinical Neurosciences, Neurology, University of Helsinki and Helsinki University HospitalHelsinki, Finland

**Keywords:** paired associative stimulation, transcranial magnetic stimulation, plasticity, spinal cord, peripheral electrical stimulation

## Abstract

**Background**: In spinal paired associative stimulation (PAS), orthodromic and antidromic volleys elicited by transcranial magnetic stimulation (TMS) and peripheral nerve stimulation (PNS) coincide at corticomotoneuronal synapses at the spinal cord. The interstimulus interval (ISI) between TMS and PNS determines whether PAS leads to motor-evoked potential (MEP) potentiation or depression. PAS applied as a long-term treatment for neurological patients might alter conduction of neural fibers over time. Moreover, measurements of motoneuron conductance for determination of ISIs may be challenging in these patients.

**Results**: We sought to design a PAS protocol to induce MEP potentiation at wide range of ISIs. We tested PAS consisting of high-intensity (100% stimulator output, SO) TMS and high-frequency (50 Hz) PNS in five subjects at five different ISIs. Our protocol induced potentiation of MEP amplitudes in all subjects at all tested intervals. TMS and PNS alone did not result in MEP potentiation. The variant of PAS protocol described here does not require exact adjustment of ISIs in order to achieve effective potentiation of MEPs.

**Conclusions**: This variant of PAS might be feasible as a long-term treatment in rehabilitation of neurological patients.

## Introduction

Paired associative stimulation (PAS) is a technique where transcranial magnetic stimulation (TMS) is synchronized with peripheral nerve electrical stimulation (PNS); signals are timed to coincide at synapses at cortical (Stefan et al., 2000) level to enhance corticospinal excitability. In spinal PAS, signals are timed to coincide at the spinal cord level (Taylor and Martin, 2009; Cortes et al., 2011; Leukel et al., 2012; Shulga et al., 2015). PAS protocol typically consists of a single TMS pulse combined either with a single PNS pulse or 10-Hz PNS trains (Carson and Kennedy, 2013).

These conventional protocols lead to long-term potentiation (LTP)-like effect at a limited range of interstimulus interval (ISIs; Bunday and Perez, 2012; Carson and Kennedy, 2013; Shulga et al., 2015). If PAS would be applied in multiple sessions to enhance corticospinal connections as a tool for long-term rehabilitation, the initially calculated ISI would need to be adjusted constantly, as neuronal conductivity may change over time during recovery of the injury. Moreover, it is plausible that the remaining neural pathways in patients with neurological diseases have a wide range of conductivities as a result of partial injuries; precise measurements of conductivities in such patients can be challenging.

Whereas PAS protocols with single peripheral pulses are best suited for studying plasticity mechanisms, more clinically feasible types of PAS protocols are required for medical treatment development. Such protocol should reliably enhance corticospinal transmission at a wide range of ISIs. One possibility to design such protocol is to increase the number of interactions between pre- and postsynaptic volleys through the increase of volley number: when LTP-inducing and LTD-inducing timing interactions occur at the same time, LTP can override LTD (Sjöström et al., 2001). The increase in the number of orthodromic volleys can be achieved by increasing TMS intensity; high-intensity TMS pulses result in a high-frequency repetitive discharge of corticospinal neurons (Di Lazzaro et al., 2008). To increase the number of antidromic volleys, high-frequency trains of PNS can be used.

The potential of single-session spinal PAS as rehabilitation for spinal cord injury patients, and of multiple-session cortical PAS for stroke patients has been reported (Uy et al., 2003; Bunday and Perez, 2012). We have recently published a case report of two incomplete spinal cord injury patients showing the positive long-term effect of multiple-session spinal PAS consisting of single high-intensity TMS pulses combined with 50 Hz PNS trains (Shulga et al., 2016). Here we characterize single-session PAS with these parameters in healthy subjects. We show that this PAS protocol reliably leads to potentiation of motor-evoked potential (MEP) amplitudes at a wide range of ISIs.

## Materials and Methods

### Subjects

Five subjects (3 males; right-handed; age 30–60, mean age 40) participated in the study. Each subject participated in 10 experiments, conducted on different days. The study was conducted as a part of clinical trial approved by Helsinki University Central Hospital medical ethical committee; a written informed consent was obtained from each subject.

### Determination of Interstimulus Interval

We determined ISIs 0 ms (antidromic and orthodromic pulses arriving simultaneously at corticomotoneuronal synapses at the spinal cord level) between TMS pulse and the first pulse of the peripheral train with the formula F latency minus MEP latency, as described previously (Shulga et al., 2015). We used minimal F-latency (the shortest latency in a series of 10 measurements) at supramaximal stimulation (the stimulation intensity at which increasing the intensity does not further produce the increase in F wave amplitude) with 0.2 ms pulses. MEPs were elicited by TMS at 120% of resting motor threshold (RMT). RMT was determined as the minimum intensity required to produce a MEP over 50 μV in over 50% times in a series of 10 pulses.

### Navigated Transcranial Magnetic Stimulation

TMS was given with eXimia magnetic stimulator (Nexstim Ltd, Helsinki, Finland). For stimulation during PAS session, TMS was given at 100% SO; for measurement of MEPs, TMS was given at 120% RMT (see above). We used figure-of eight coil; the outer diameter of the coil was 70 mm. The induced current was oriented perpendicular to the sulcus at the stimulation target at the beginning of the mapping. The optimal site for TMS was determined individually for each subject based on the mapping of the motor cortex: the spot and coil orientation most readily eliciting MEPs from abductor hallucis muscle was selected. The selected spots and coil orientation were registered in our MRI-guided navigation system (NBS navigation system, Nexstim Ltd, Helsinki, Finland). The navigation system ensured that the TMS stimulation target and coil orientation of the PAS protocol was the same as the stimulation site used for MEP measurements.

### Peripheral Electrical Stimulation

We delivered PNS using Dantec Keypoint^®^ electroneuromyography device (Natus Medical Incorporated, Pleasanton, CA, USA). Peripheral electric stimulation was delivered as 50 Hz trains of 1-ms square wave pulses for 100 ms (= 6 pulses per train) to depolarize lower motor neurons’ somata and dendrites by antidromic motor neuron volleys. The individual minimum intensity required to produce F-responses when measured with single 1-ms pulses was used for the 50-Hz trains.

Two subjects (2 and 3) perceived peripheral stimulation as unpleasant. Their skin was locally anesthetized prior to the experiments with 2.5% lidocaine/prilocaine ointment (EMLA^®^). EMLA penetrates 3–5 mm into the skin (Gajraj et al., 1994) and thus does not affect the conductivity of the tibial nerve. Parameters used for each subject are presented in Table 1.

**Table 1:** **Settings used for each subject**.

Subject	RMT (% of SO)	PNS intensity (mA)	EMLA	F latency	MEP latency (ms)	ISI 0 ms (ms)	ISI −5 ms (ms)	ISI +5 ms (ms)	ISI −10 ms (ms)	ISI +10 ms (ms)
**1**	83	11	−	50	42	8	3	13	−2	18
**2**	52	5	+	48	43	5	0	10	−5	15
**3**	64	9	+	58	48	10	5	15	0	20
**4**	61	20	−	50	48	2	−3	7	−8	12
**5**	61	6	−	52	43	9	4	14	−1	19

*SO—stimulator output, Positive ISI—PNS precedes TMS, negative ISI—TMS precedes PNS*.

### Paired Associative Stimulation

Both peripheral stimulation and TMS were triggered by Presentation^®^ software (Neurobehavioral Systems Inc., Albany, NY, USA) to guarantee the adjusted ISIs. The peripheral electric stimulation train was delivered once every 5 s, each train synchronized with single-pulse TMS, for 20 min. All experiments were applied to left motor cortex/right tibial nerve.

During the stimulations (PAS or PNS only), subjects were instructed to mentally concentrate on the movement produced by electrical stimulation of the tibial nerve (toes flexion of the right leg), but not to attempt to move the leg or toes during the stimulation.

### Measurements of MEPs

**Figure 1:**
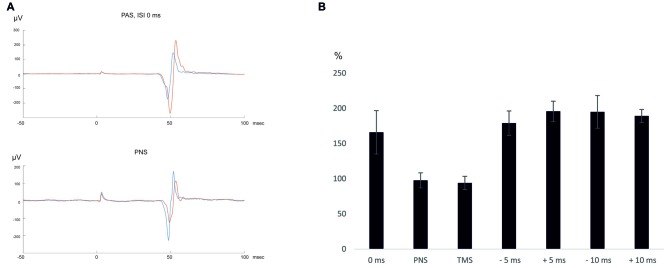
**(A)** An average of 10 motor-evoked potentials (MEPs) recorded in a representative subject (subject 2). Blue—pre-paired associative stimulation (PAS)/peripheral nerve stimulation (PNS), red—post-PAS/PNS. **(B)** Summary of the results.

Ten MEPs were measured at 120% RMT, as described above, immediately before and after the stimulation. For graphical presentation of the results (Figure 1), the MEP traces were averaged using MATLAB (MathWorks Ltd., Nattick, MA, USA) software.

### Statistics

To compare the averages of MEP amplitudes pre-PAS vs. post-PAS, we used Wilcoxon signed ranks test. To investigate whether there is a difference between PAS groups with different ISIs, we used Kruskal-Wallis test. Statistical tests were done on IBM SPSS Statistics 22 Software. Data is presented as mean ± standard error.

## Results

Each subject received PAS sessions at intervals 0 ms, ±5 ms and ±10 ms as well as 300 ms, one PNS only, and one TMS only session. The detailed results are presented in Table 2.

**Table 2:** **Detailed results shown separately for each subject**.

	MEP amplitude, μv		% post/pre		MEP amplitude, μv		% post/pre
Subject	pre	post			pre	post
	**PAS, ISI 0 ms**				**PNS only**		

1	281	804	**286**		254	287	**113**
2	369	582	**158**		211	141	**67**
3	161	210	**130**		85	68	**80**
4	659	931	**141**		665	840	**126**
5	900	1032	**115**		700	710	**102**
**Average**			**166 ± 31%**				**98 ± 11%**

	**PAS, ISI +5 ms**				**PAS, ISI −5 ms**		

1	257	603	**235**		540	970	**180**
2	277	587	**211**		360	640	**178**
3	102	192	**189**		91	121	**134**
4	864	1279	**148**		681	1111	**163**
5	952	1869	**196**		370	888	**240**
**Average**			**196 ± 14%**				**179 ± 17%**

	**PAS, ISI +10 ms**				**PAS, ISI −10 ms**		

1	471	886	**188**		337	740	**220**
2	433	883	**204**		117	261	**222**
3	140	295	**211**		75	188	**251**
4	959	1504	**157**		899	1121	**125**
5	394	734	**186**		447	703	**157**
**Average**			**189 ± 9%**				**195 ± 23%**

	**TMS only**				**PAS, ISI 0 ms, no motor imagery**		

1	248	238	**96**		247	371	**151**
2	275	294	**107**		302	666	**220**
3	75	69	**91**		64	178	**277**
4	319	193	**60**		614	743	**121**
5	498	578	**116**		648	1455	**224**
**Average**			**94 ± 10%**				**199 ± 28%**

	**PAS, ISI 0 ms, 30 min and 60 min follow-up**						

	**MEP amplitude, μv**				**% of pre-PAS**		

**Subject**	**pre**	**post**	**30 min**	**60 min**	**post**	**30 min**	**60 min**

1	281	804	1102	725	**286**	**393**	**258**
2	369	582	588	455	**158**	**159**	**123**
3	67	216	61	53	**325**	**91**	**80**
4	695	972	793	790	**140**	**114**	**114**
5	627	1062	1026	767	**170**	**164**	**122**
**Average**					**216 ± 42%**	**184 ± 54%**	**139 ± 31%**

	**PAS, ISI 300 ms**		
	**MEP amplitude, μv**		**% post/pre**

**Subject**	**pre**	**post**

1	271	298	**110**
2	322	246	**76**
3	94	263	**280**
4	1050	932	**88**
5	258	595	**230**
**Average**			**157 ± 41%**

After PAS with 0 ms ISI, MEP amplitudes increased to 166 ± 31% (*p* = 0.043 post vs. pre- PAS), whereas after PNS only, there was no change in MEP amplitudes (98 ± 11% post vs. pre-PNS, *p* = 0.69). TMS only did not induce MEP amplitude potentiation either (94 ± 10% post vs. pre-TMS, *p* = 0.89). MEP latencies did not change after PAS (post-PAS minus pre-PAS: 0.1 ± 0.4 ms, *p* = 0.69) and after TMS (post-TMS minus pre-TMS: 0.88 ± 0.82 ms, *p* = 0.08) and became slightly prolonged after PNS (post-PNS minus pre-PNS: 1.1 ± 0.3 ms, *p* = 0.043). At 30 min and 60 min after PAS with 0 ms ISI, there was a trend towards higher MEP amplitudes (184 ± 54%, *p* = 0.08, at 30 min and 139 ± 31%, *p* = 0.08, at 60 min); the potentiation persisted at least for 60 min in four out of five subjects (see Table 2).

PAS increased MEP amplitudes at all tested intervals: +5 ms (196 ± 14%, *p* = 0.043), −5 ms (180 ± 17%, *p* = 0.043), +10 ms (189 ± 9%, *p* = 0.043) and –10 ms (195 ± 23%, *p* = 0.043). The MEP latencies did not change (post-PAS minus pre-PAS, +5 ms ISI: 0.6 ± 0.5, *p* = 0.18; −5 ms ISI: −0.1 ± 0.2, *p* = 0.5; +10 ms: −0.9 ± 0.7 ms, *p* = 0.23; −10 ms: −0.3 ± 0.1 ms, *p* = 0.08). Although MEP amplitude increase at ±5 ms and ±10 ms ISI was larger than at 0 ms ISI (Table 2), amplitude increases did not differ significantly between the groups (*p* = 0.58 by Kruskal-Wallis test). In order to test an interval where TMS and PNS-induced volleys are not timed to arrive simultaneously, we applied PAS at ISI 300 ms (that is, TMS coming 200 ms after the last PNS pulse); at this interval, there was no significant increase in MEP amplitudes (157 ± 41%, *p* = 0.5).

All experiments mentioned above were conducted in the presence of motor imagery (see “Materials and Methods” Section). We conducted a separate experiment without motor imagery at ISI 0 ms; PAS induced MEP potentiation also without motor imagery (198 ± 28%, *p* = 0.043, Table 2).

## Discussion

Our PAS protocol reliably induced MEP potentiation at a wide range of ISIs in a robust way in healthy subjects, and might thus be more suitable for clinical use than conventional PAS protocols. The conventional PAS protocol has a single-pulse TMS and PNS; its properties have been investigated in detail (Carson and Kennedy, 2013). In this work, we wanted to present a new variant of PAS protocol. We have previously examined the properties of another variant of conventional PAS which utilized 10 Hz PNS (Shulga et al., 2015) with the same equipment and ISI determination as presented here; this protocol did not induce MEP potentiation at ±10 ms ISI. TMS pulses were given at maximum intensity to ensure multiple collisions of pre- and postsynaptic pulses at the spinal cord level. In neurological patients, upper motor neurons might be less excitable than in healthy subjects, and high-intensity TMS would ensure their excitation.

For PNS, we used high-frequency peripheral pulses at minimal intensity required to elicit F-response to ensure the activation of the motor neurons. We have previously reported the use of otherwise identical protocol in one healthy subject at intensity higher than minimal intensity for F-response (15 mA); this has led to MEP potentiation observable at 1 h, but not immediately after PAS (Shulga et al., 2016). Lowering the intensity in the same subject to 11 mA (subject 1 in this study) has led to observable MEP potentiation immediately after the protocol. It is probable that both intensities enhance corticospinal transmission immediately, but higher intensity leads to exhaustion of the muscle which covers the effect.

The ensuing peripheral stimulation was unpleasant for some subjects. The use of EMLA ointment reduced these unpleasant sensations. Gradual adaptation to the peripheral stimulation during subsequent sessions was also reported. In neurological patients, peripheral nerves can be less excitable than in healthy subjects, and even higher stimulation intensities might be required. The possibility to use EMLA as well as the adaptation to the unpleasant properties of the PNS during the sessions should be kept in mind when designing PAS protocols for these patients.

The use of TMS or PNS alone did not lead to potentiation of MEP amplitudes. The control experiment where we applied TMS 300 ms after first PNS pulse (that is, 200 ms after the last PNS pulse) resulted at a group level in MEP potentiation within the same range as other tested intervals. The result was, however, highly variable between the subjects as both decreases and increases of MEPs were observed, and not significant (see Table 2). It is plausible that depolarization of lower motor neurons induced by PNS has different duration in different subjects. When TMS and PNS are timed to coincide closely on spinal cord, and possibly also cortical, level, there is a stable MEP amplitude increase in all subjects. However, when PNS and TMS pulses are as far as 200 ms apart, there is an interaction between antidromic and orthodromic depolarization only in part of the subjects.

The use of high-frequency PNS naturally hampers the interpretation of the neural level of the physiological effects, as the relation between the TMS- and PNS-induced activations gets more complicated. In the protocol presented here, the arrival timing between the pre-and postsynaptic volleys at the spinal cord level is way much closer than possible interactions occurring at the cortical level; it is thus plausible that observed MEP potentiation originates mainly from induction of plasticity at corticomotoneuronal synapses of the spinal cord. However, it is plausible that also somatosensory afferents are activated, and interactions occur at multiple levels. Further research is needed to elucidate the mechanism of MEP amplitude potentiation by the protocol presented here more precisely.

We conducted all experiments in the presence of motor imagery, since we used this method on our spinal cord injury patients (Shulga et al., 2016). Control experiment without motor imagery revealed that it is not required for MEP amplitude potentiation in healthy subjects at 0 ms ISI. However, this observation should be interpreted with caution when designing the protocols for neurological patients: in these patients, RMTs can be much higher than in healthy subjects, and lowering RMT through motor imagery might be helpful to achieve the desired effect of PAS.

Spike-time dependent plasticity (STDP) is dependent on numerous factors: the firing rate, the number of coactive synaptic inputs, the postsynaptic voltage and the timing of the inputs, among others (Feldman, 2012). Importantly for this protocol, it has been shown *in vitro* that spike-timing relationships causing LTP can “win” out over those favoring LTD when multiple interactions occur at the same time (Sjöström et al., 2001).

## Conclusion

Our aim was to develop and test tools for patient rehabilitation, and the observed increase of efficacy of the motor activation by the PAS is promising in this respect. The results support the usefulness of the stimulation parameters selected for our proof of concept—study of PAS used in rehabilitation of two patients with spinal cord injuries (Shulga et al., 2016).

## Author Contributions

AS, AZ, PL and JPM: conception and design of the work, acquisition, analysis, and interpretation of data for the work, drafting the work and revising it critically for important intellectual content, final approval of the version to be published, agreement to be accountable for all aspects of the work.

## Funding

This study was supported in part by research grants from the Faculty of Medicine, University of Helsinki, Emil Aaltonen Foundation and Finnish Cultural Foundation.

## Conflict of Interest Statement

PL reports personal fees from Nexstim Ltd, outside the submitted work. Other authors have nothing to disclose.
